# Supplementary material for: Are carbon emissions trading and green financial instruments synergistic? -Comprehensive quantitative research based on content analysis
Source: PLoS One. 2024 Mar 7;19(3):e0298601. doi: 10.1371/journal.pone.0298601 (PMC10919601; doi:10.1371/journal.pone.0298601)
Supplement: S1 Data — (ZIP) [file pone.0298601.s001.zip › Data/地级市Z_F工作报告.zip!/╝¬┴╓/═¿╗»╩╨/└┤╫╘╣1⁄2╓┌║┼í╛┬φ┐╦╩2╛▌═°í┐_2010.docx]

一、2009年工作回顾

　　2009年，是通化发展遇到巨大困难和严峻挑战的一年。在市委的正确领导下，在市人大、市政协的监督支持下，市政府团结带领全市人民，全力以赴保增长、保民生、保稳定，圆满完成了市六届人大二次会议确定的目标任务。预计：

　　——地区生产总值实现513.5亿元，增长15.1%。

　　——全口径财政收入实现47.2亿元，增长0.5%，其中地方级财政收入实现26亿元，增长21.4%。

　　——规模以上工业增加值实现220亿元，增长15.5%。

　　——固定资产投资完成605亿元，增长40.9%。

　　——招商引资到位资金510亿元，增长28%；实际利用外资1亿美元，增长15.2%。

　　——社会消费品零售总额实现207亿元，增长21%。

　　——城镇居民人均可支配收入15500元，增长11.2%；农民人均纯收入5730元，增长11.7%。

　　过去的一年，我们着力推进经济社会平稳较快发展，妥善处理关系发展稳定的大事难事，主要做了以下工作：

　　坚持投资拉动不动摇，项目建设和招商引资成果显著。实施各类项目1910项，其中新开工项目1675项、3000万元以上项目610项，新储备超亿元后续项目651项，项目数量和质量创历史最好水平。开工建设了100万吨还原铁、生物疫苗、“两渣一灰”等165个亿元以上项目，20万吨啤酒、45万吨特优级食用酒精等一批重点项目建成投产。积极争取国家扩大内需政策支持，172个项目列入国家投资计划，累计到位资金11.7亿元。招商引资再结硕果，全市引进1000万元以上项目1200项，其中亿元以上项目190项，吉林亚泰、江苏高力、广东康美、浙江义乌国际小商品城等知名企业落户我市。同丹东市签署了合作协议，建设通化陆港和通丹经济开发开放先导区进入启动阶段。通化经济开发区基础设施和项目建设扎实推进，引进和投产1000万元以上新项目41项，生产总值、固定资产投资分别增长50%、62%。7个县（市、区）开发区发展壮大，形成了初具规模的项目集群。

　　强化工业立市、产业兴市，工业经济发展态势逐步趋好。采取政企联手、银企配合、产需衔接、要素保障等一系列措施，推动工业运行企稳回升。预计规模以上工业总产值、增加值增速分别比年初回升26.1和25.4个百分点。钢铁产业结构升级继续推进，通钢新一轮技术升级改造工程全面启动，3万吨减振复合钢板等一批围钢项目陆续上马。全力支持通钢稳定发展，通钢生产经营形势总体平稳。医药、食品产业保持强势增长，“通化高技术产业基地”纳入国家东北振兴战略布局，7000万支人胰岛素针剂、人参产业基地、农副产品加工示范园区等重大项目建设和产品开发、产业整合、企业并购取得积极进展，修正、东宝、万通、阜康等重点企业产出能力进一步增强。能源、新型建材、石油机械和其他优势产业都实现了较快发展。组织银企对接12次，签约融资金额46亿元。中小企业成长工程深入实施，新增规模以上工业企业86户。开工技改项目403项，开发新产品110种。民营经济主营业务收入增长23%、实缴税金增长20.5%。节能减排完成了省下达的任务。

　　狠抓基础设施建设，城市功能和人居环境明显改善。通丹铁路全线开工，通丹高速公路完成路基主体80%，梅沈高速公路完成路基工程95%，营松高速公路建设加快推进，通长、通集高速公路前期工作取得进展，通化机场正式奠基。

　　年初确定的市区十项城建重点工程全面完成。二道河改造工程如期竣工，沿河环境和配套设施彻底改善，防洪标准整体提高。污水处理工程进展顺利。完成了2.9公里滨江东路和光明大街等20条主次街路改造任务，维修加固了红旗桥。江南新区基础设施建设步伐加快。解决了城区周边1.9万人吃水和4000户居民用气问题，改造了35栋老旧楼房供热设施。综合整治旧住宅小区9万平方米。群众反映强烈的排水设施堵塞等问题得到解决。市区拆迁棚户区40万平方米，开工建筑面积73.8万平方米；5个县（市）拆迁棚户区34万平方米，开工建筑面积109万平方米。完成了城市总体规划纲要和土地利用总体规划修编，强化城市环境综合整治和管理，提升江西、完善江东、开发江南、建设江北取得突破性进展。

　　推进改革创新和服务业升级，发展活力进一步增强。处理国企改制遗留问题取得新进展，57户国有关闭破产企业退休人员全部纳入城镇职工基本医疗保险。集体林权制度主体改革成效明显，配套改革试点全面推开。粮食清仓查库和国有粮食购销企业改革顺利完成。收储土地695公顷，招拍挂出让土地97公顷。与7家省级金融机构实现战略性合作，争取三年授信额度720亿元；组建了11户小额贷款公司，成立了城建投资集团；通天酒业在香港成功上市。实施旅游建设项目57项，高句丽遗迹入选“吉林八景”，旅游业收入增长38%。新天地购物公园、长白山医药物流、龙湾会馆等重点商贸项目快速推进，义乌国际商贸城、恒坤家居博览购物中心即将开业。

　　深入实施县域突破战略，新农村建设水平整体跃升。县域发展保持了争先晋位的强劲势头，工业化、城镇化步伐加快。开工1000万元以上农业产业化项目38项，省级以上龙头企业发展到46户，农产品加工量突破190万吨。新建标准化牧业小区130个，畜牧业产值增长23.3%。绿色有机无公害食品标识增加到240个。植树造林27.3万亩，实现了连续55年无重大森林火灾。农村公路竣工里程831公里，通屯率达到78.1%。完成了浑江干流整治等52项水利设施建设项目。克服严重干旱影响，粮食总产量达到35亿斤。新农村建设整体推进，12个镇列入全省“百镇建设工程”，其中4个镇成为首批启动镇，80%的行政村达到“四化四有”标准。全省县域突破工作现场会和百镇建设工程现场会暨启动大会先后在我市召开。通化市获得全省县域突破组织推动奖，梅河口市、集安市获得升级晋位奖，柳河县获得薄弱县（市）争先跨越奖，通化县、辉南县获得单项奖。

　　统筹经济社会发展，社会事业取得新进步。列入省科技计划项目33项，获得省级科技进步奖励项目5项。东昌区被评为省内第一个国家科技进步示范区。基础教育健康发展，教育基础设施、校园环境得到较大改善。通化技师学院挂牌成立，职教园区一期工程投入使用。国庆60周年系列活动蓬勃开展，广场文化、社区文化、农村文化丰富多彩。设立了文化产业发展扶持资金，8个项目列入省第二批非物质文化遗产名录，高句丽文物展示中心、松花石（砚）交易展览中心、人参文化展览馆建成使用。全民健身活动广泛开展，竞技体育获国家级金牌13枚、省级金牌75枚。公共卫生和医疗服务体系更趋完善，甲型H1N1流感等传染病防控工作扎实有效。广电事业加快发展。人口增长控制在计划目标以内。公民思想道德建设、文明和谐创建工作得到加强，城乡文明程度明显提高。民族宗教、妇女儿童、老龄、残疾人事业进一步发展，“双拥”、“双支”和“五边”建设等工作都取得了新成绩。对口支援地震灾区恢复重建任务圆满完成。援藏工作取得新成效。

　　高度重视民生工作，涉及群众切身利益的问题得到有效解决。年初承诺的十件民生实事全面完成。城镇新增就业4.7万人，农村劳动力转移就业26万人。养老、失业、医疗、工伤和生育保险新增参保28.6万人，企业退休人员人均基本养老金标准增至997元，新型农村合作医疗人均筹资标准增至100元，城乡低保补助水平进一步提高。改造农村泥草房6414户，提前两年超额完成省下达的改造任务。建成廉租房6.5万平方米，为市区1.86万户低保无房户和低收入住房困难家庭发放了租赁补贴。改造中小学危旧校舍10.9万平方米。市中医院异地建设工程开工。市儿童福利院工程竣工。建成农村饮水安全工程324处，19.2万农村人口受益。实施了哈泥河流域污染整治和水库加固清淤工程，哈泥自然保护区晋升为国家级保护区。市区空气质量优良天数达到310天。

　　切实改进政风，公共服务和社会管理工作进一步加强。主动接受人大法律监督、工作监督和政协民主监督，办理人大代表建议181件，政协委员提案154件。深入学习实践科学发展观，组织开展了以精细化管理为主题的“查找改”活动，并取得阶段性成果。聘请了第一批37名市政府义务监督员，在50个社区设立了公共服务公示板。完成了政府机构改革任务。认真整治和建设软环境，加强廉政建设，切实纠正损害群众利益的不正之风。行政审批项目调减幅度达到36%，495项审批职能进入政务大厅集中审批。市长公开电话受理群众来电4.7万件，办复率达到99%。“五五”普法整体推进。食品安全和人参、葡萄酒市场专项整治取得阶段性成效。狠抓安全生产，加大社会治安综合治理力度，着力解决信访突出问题，圆满完成国庆安保任务，打黑除恶取得新战果，维护了社会和谐稳定。

　　在过去的一年里，通化被国家认定为首批新型工业化产业示范基地，被评为中国十佳休闲宜居生态城市、十佳投资创业城市、十佳宜游城市，钢铁、医药产业入选中国百佳产业集群。

　　各位代表！在特殊的经济环境和特别困难的情况下，过去一年能够取得这些成绩，确实来之不易。这是市委正确领导的结果，是市人大、市政协监督支持的结果，是全市人民团结奋斗的结果，是社会各界共同参与的结果。在此，我代表市政府，向辛勤工作在各行各业的全市人民，向给予政府工作大力支持的人大代表和政协委员，向各民主党派、工商联、无党派人士和人民团体，向驻通国省属单位、驻通部队和武警官兵，向所有关心支持通化发展的同志们、朋友们，表示衷心的感谢！

　　在肯定成绩的同时，我们也清醒地看到，我市经济社会发展和政府工作还存在不少矛盾和问题。主要是经济发展总体还没有走出困境，以通钢为龙头的冶金产业受危机冲击较重，整个工业经济低速运行、回升不快；经济增长后劲还显不足，缺少大项目特别是工业大项目带动；社会事业基础设施建设滞后，城市功能需要尽快完善提升；保障和改善民生任务繁重，扩大就业、居民增收、社会保障、安全生产压力较大，还有一些群众生活困难；一些深层次矛盾长期积累、相互交织并开始集中显现，给社会带来不稳定因素；社会管理存在薄弱环节，环境卫生、市容市貌、物业管理需要进一步加强；软环境建设仍不尽人意，有些公职人员责任意识、服务意识不强，创新办法不多，工作作风不实，效率不高，不作为、“中梗阻”问题还较突出。这些问题，我们要采取有效措施，认真加以解决。

　　二、2010年目标任务

　　2010年是实施“十一五”规划的最后一年。做好今年工作，事关承前启后、继往开来，意义重大。当前，金融危机影响还在持续，经济发展仍然存在许多不稳定、不确定因素。我市经济自去年下半年开始企稳回升，但回升的基础还不稳固、不平衡，冶金产业回升的态势还不够明朗，走出低谷、争先晋位面临很大压力。与此同时，我国保持宏观经济政策的连续性和稳定性，为我市继续扩大投资、保持经济平稳较快发展创造了极为有利的外部条件；国务院进一步实施东北老工业基地振兴战略若干意见的出台，长吉图开发开放先导区上升为国家战略，为我市加快开发开放、加速产业升级提供了新的机遇；我市近些年不断加大固定资产投资力度，重点产业建设、开放通道建设、中心城市建设、开发区建设、县域发展和新农村建设等全面取得重大进展，为振兴崛起积蓄了能量。我们一定要认清和把握形势，切实增强“等不起”的忧患感、“慢不得”的危机感、“坐不住”的紧迫感，坚定必胜信心，勇于任事，敢于担当，突破困境，为赢得“十二五”发展的高起点开局奠定坚实基础。

　　2010年市政府工作的基本思路是：认真贯彻党的十七届四中全会和市委五届五次全会精神，深入落实科学发展观，坚持保增长、保民生、保稳定，走产业规模化、交通快速化、人居生态化发展之路，强力推进固定资产投资上规模，在大项目建设和产能释放、达产达效上求突破；强力推进十大产业建设，在产业跃升上求突破；强力推进中心城市崛起，在构建开发开放新格局上求突破；强力推进县域发展，在新农村建设上求突破；强力推进民生改善和社会事业发展，在和谐通化建设上求突破，加快建设东北东部地区中心城市和中国北方宜居山水城市，为通化振兴崛起而奋斗。

　　主要预期目标：地区生产总值增长15%左右，规模以上工业增加值增长15%，全口径财政收入增长16.5%，社会消费品零售总额增长20%以上，城镇居民人均可支配收入、农民人均纯收入分别增长10%以上，城镇登记失业率控制在4.2%以内，人口自然增长率控制在3.1‰以内。

　　完成今年主要指标，要在具有影响和支撑作用的重点工作上取得突破。全年固定资产投资完成800亿元以上，重点建设100个亿元以上重大工业项目，实施100个重点技术改造项目，抓好100个重点工业项目投产达效，扶持100户重点企业，新增100户规模以上企业。

　　（一）集中力量抓项目、抓招商。继续组织开展“项目建设年”活动，进一步强化投资拉动和项目带动，突出抓好“四大工程”、“六大板块”项目建设。规划实施各类项目1771项，其中新建项目1381项、3000万元以上项目626项、亿元以上项目202项。开工建设新2号高炉、2万吨镁合金等24个10亿元以上重大项目，100万吨还原铁、80万吨焦化等重点项目全面建成投产。加大项目和资金争取力度，充分利用全国资源型城市转型试点政策，推进经济转型和城市转型。系统做好项目谋划特别是项目前期工作，夯实项目接续基础。保障项目用地，节约、集约、规范使用土地，提高投资强度。创新方式，凝聚合力，提高招商引资的实效性。全年引进资金600亿元，实际利用外资1.2亿美元。依托重点产业和资源优势，积极引进产业集聚、产业链延伸项目和资源开发、精深加工型企业。加快招商平台建设，推动开发区加快发展。通化经济开发区基本完成湾湾川和冶金、现代装备制造产业园等中心区域基础设施规划建设，落地、开工和投产亿元以上项目30项。7个县（市、区）开发区基本形成产业规模，成为县域发展的重要支撑。着力破解金融难题，敢于和善于融资，不失时机筹措资金，构建多层次资本市场体系。全力争取金融机构和政策性银行信贷支持,加快BOT、BT、TOT、PE等模式的实际应用。做好企业上市工作，扩大股权融资。树立经营城市理念，与再担保、金融机构、各类基金等战略投资者创新合作，鼓励民间投资。

　　（二）强力推动工业结构调整优化。围绕建设“十大产业”、“三大基地”和四个百亿元级产业集群，实施工业产业跃升计划。冶金产业，全力支持通钢战略重组和技术升级改造，加快1000万吨钢产能建设，推进20个重点围钢项目建成投产，规划建设汽车零部件制造中心，提高钢材就地转化率和制品化率。医药产业，重点实施修正60亿粒空心胶囊、万通2500万支脑蛋白等68个项目，推进基因重组人胰岛素系列生物制品产业化，拓展中药制剂、中药保健品、医疗器械、药品包装等生产领域。食品产业，抓好25万吨有机米、万吨大泉源优质酒等121个项目，着力在产品的系列化、品牌化开发上求突破。葡萄酒产业，发挥通化葡萄酒、通天酒业两户上市公司带动作用，支持重点企业整合重组、开发市场和经营模式创新，加快通化葡萄酒品牌振兴。机械制造产业，全面提升研发设计、加工制造和系统集成整体水平，推动产业向集约化、信息化和国际化方向发展。建成国家级石油机械检测中心。建材、化工、信息等产业都要谋划和新上一批大项目。加强铁矿、煤炭、松花石、石灰石等资源的勘探开发和保护，完成第二轮矿产资源规划修编，发展优势资源产业。切实加强现有企业的挖潜改造、资源整合和自主创新，加速装备、技术和产品全面升级，落实产能扩张和效益倍增计划，促进扩大规模、加快发展。坚持实施品牌战略，广泛开展质量兴市活动，培育和创造一批具有通化特色的名牌产品、名牌企业。加强医药、冶金、长白山特产品出口基地和出口平台建设，推动外贸出口稳定增长。加强节能减排工作，完成省下达的指标任务。强化工业运行组织和协调服务，搞好产需衔接、银企对接，组织企业开展管理创新，保障生产要素供应，力保工业快速回升、平稳运行。

　　（三）大力加强基础设施建设。着眼东北东部加快开发开放新态势，启动实施通丹经济开发开放先导区规划，在更高平台上推动与长吉图开发开放先导区、辽宁沿海经济带深度接轨，逐步把通化建设成为面向周边国家和环渤海经济圈的开放前沿。着力抓好十项重大基础设施工程。一是公路工程。加快通丹、营松、梅沈高速公路建设进度，开工通化至靖宇高速公路。做好通长、通集高速公路前期工作。二是铁路工程。全线推进通丹铁路建设，完成全部桥涵及隧道工程。筹划高速铁路和沈吉复线建设项目。三是机场工程。完成通化机场主跑道和部分配套设施建设，规划建设三源浦空港经济区和机场沿线基础设施。四是陆港工程。开工建设具有相应保税功能，集服务、联检、物流为一体的通化陆港。五是电力工程。开工建设2×30万千瓦煤矸石热电联产和望江楼、文岳水电站及城乡电网等一批重点电力项目。六是能源工程。开工梅河煤矿500万吨扩能改造工程，加快油页岩、抽水蓄能等重大项目推进节奏。七是水利工程。继续实施浑江干流整治，新建长胜至二道江大桥2.3公里防洪江堤工程，抓好病险水库除险加固、灌区配套和节水改造等48个水利项目。八是城市公共设施工程。规划实施地下管网改造和市政设施升级晋档，推进周山沟河整治和架空线路落地，筹建第二水源和垃圾无害化处理厂，建成医疗垃圾处理厂。九是旧城改造工程。重点推进江东、江西和城区周边改造，支持二道江区旧城改造和矿区环境治理、污染治理。十是新区建设工程。新建续建江南6条路网和水热气等基础设施，加快江南开发及业态提升。

　　（四）扎实推进农村工业化、城镇化和农业现代化。围绕人参、中药材、绿优米、葡萄、林蛙、食用菌等特色资源，加强科技攻关和政策扶持，积极引进有实力的大企业，实施深度开发和综合利用，提质量、创品牌、增效益。突出抓好5个亿元以上、10个3000万元以上农业产业化项目建设，争取更多农业和农村项目进入全省“五个一百工程”。推动重点龙头企业加快发展，农产品加工能力达到220万吨。调整优化种植结构，优质特用粮食种植面积突破330万亩。打好畜牧业发展攻坚战，新建2个牧业产业园区和130个标准化牧业小区，牧业产值增长21%。完成集体林权配套改革，抓好林业资源保护和木材加工园区等产业工程，壮大林业经济。继续抓好森林防火。扩大棚膜经济等特产业生产规模，园艺特产业产值增长10%。推行标准化生产，完善检验检测体系，确保农产品质量安全。推动农村宅基地规范化治理和土地承包经营权流转。加强金融、科技、信息等支农服务。启动“百村示范、全面提升”工程，抓好100个新农村建设示范村，整体推进以“四清、四改、两化”为主的环境综合整治，继续改善村容村貌。加快实施“百镇建设工程”，重点加强列入全省规划的启动镇建设，培育经济强镇，快速发展县城和区域中心镇，逐步打造一批特色鲜明、经济发达、功能齐全、环境优美的强镇、重镇和名镇。

　　（五）全面提升城市规划、建设和管理水平。坚持高标准规划、高质量建设、精细化管理，突出宜居特色，做好山水文章，进一步提升城市品位。

　　编制完成城市总体规划、重点区域控制性详细规划和景观风貌、绿化体系、道路交通等专项规划。积极探索创新城市规划建设体制机制，规划建设通化都市区，为发展百万人口城市创造条件。市区城建计划投资30亿元。加快完善城区路网，建设光明大街、东昌路两条精品路，改造提升新华大街、和平路等主次街路和二道江城区街路。建成5万吨污水处理厂，城区污水基本实现达标排放。开工集中供热三期工程。实施滨江东路佐安河口至二道江大桥段堤路改造，全线贯通沿江景观带。坚持绿化美化并举，栽植乔木、灌木100万株，新建二道河等区域精品绿化带。建设江南公园。提高滨江沿河、城区街路亮化档次，更新改造居民巷道和楼道照明设施。在浑江一期平湖下游新建跨江人行景观桥，打造新的观景平台和亲水休闲区。实施一批污染治理和生态保护工程，市区空气质量优良天数达到315天。创新办法，完善机制，全面加强城市管理。出台新的物业管理办法，提高物业管理水平。积极创建国家卫生城市和省级园林城市。

　　（六）加快民营经济腾飞和服务业跨越发展。继续推动全民创业，深入实施中小企业成长工程，搭建服务平台，拓宽融资渠道，扶持成长型企业做大做强，形成支持民营企业、尊重民营企业家、促进民营经济大发展的浓厚氛围。突出发展生产性服务业，推进长白山医药物流、长胜货物编组及河口客货转运中心等重点物流项目。加强旅游产业规划，实施45项旅游建设项目，大力开展旅游招商和宣传推介，深度开发客源市场，争创中国优秀旅游目的地。积极打造东北东部区域商贸中心、物流中心，支持欧亚、恒坤、义乌、中东、高力等知名企业在通化扩大发展规模。加快建设国际小商品配货中心、御景首府综合体、餐饮休闲文化城，建成新天地购物公园、龙湾会馆，尽快把江南新区打造成为旅游接待中心、购物中心。稳定发展商业地产和工业地产。

　　（七）协调发展各项社会事业。实施科技创新“310工程”，规划建设省级医药科技产业园，申报60项国家和省科技发展计划项目。优化教育结构布局，提高教师队伍素质，促进义务教育均衡发展。深化职业教育办学模式改革，加快实施职教园区配套工程。推进校舍安全工程，重点改善200所中小学校特别是薄弱校办学条件。完成市青少年活动中心改造项目。支持通化师院和技师学院改革创新。建立人力资源交流储备平台，广泛吸引各类人才。完善医疗卫生保障体系，支持首批200个村卫生所和社区卫生服务站建设。扩建市医院、精神病院，完成县级医院改造任务，市中医院、残疾人康复中心投入使用。抓好甲型H1N1流感等传染病防控工作。大力发展文化事业和文化产业，打造长白山地域文化中心。筹建文化产品交易市场，开工建设集科技馆、博物馆、展览馆为一体的科技文化中心，建设图书馆、档案馆，修缮高志航纪念馆。落实全民健身计划，提高竞技体育水平。加快广播电视微波数字化改造。稳定低生育水平，提高出生人口素质。深入开展文明和谐创建活动，建设现代文明城市。做好民族宗教工作。加强“双拥”、“双支”工作，巩固发展军政军民团结，争创全国双拥模范城“七连冠”。

　　（八）进一步改善民生。坚持改善民生不停步、维护稳定不放松，让人民群众共享发展振兴成果、生活安宁幸福。认真落实促进就业各项政策，创新就业岗位开发模式，加强对大学毕业生、就业困难人员和失业人员的就业服务。城镇新增就业5万人，农村劳动力转移就业27万人。抓好社会保险扩面攻坚，养老、失业、医疗保险新增参保3万人。提高企业退休人员养老金水平和城乡低保群众、优抚对象生活补助标准。统筹社会资源，完善救助机制，对城乡低收入群体实行有效帮扶救助。加快建设保障性安居工程。积极推进“暖房工程”，保障城市冬季供暖。改造农村危房5000户，建成农村饮水安全工程180处，新建120个农村文化活动室、860个农家书屋、100个健身广场、100个便民超市、30个医疗卫生室和计生室。综合整治哈泥河流域污染，实施人工湿地建设和污水处理、水库清淤工程，保障城市饮水安全。强化生产、加工和市场监管，确保食品药品安全，维护消费者合法权益。

　　集中力量办好市区十件惠民实事。棚户区及危旧房改造新建续建100万平方米；综合整治旧住宅小区100栋；新增集中供热入网面积100万平方米；改造巷路100条；新建维修公厕100座；解决城区周边3万人吃水、4000户居民用气和30栋老旧楼房采暖问题；企业退休人员人均养老金每月增加130-140元；3000户城市低保边缘户纳入社会救助体系；为1.8万户城镇低收入家庭发放住房补贴；新型农村合作医疗政府补贴增至120元，新型农村社会养老保险试点惠及农民3万人。

　　精心维护社会稳定，创建平安通化。坚持不懈地抓好安全生产，深化煤矿、道路交通、建筑施工、消防等领域隐患排查整治，加强基层基础建设，坚决遏制重特大事故发生。强化社会治安综合治理，全面推进“五项”建设，依法打击各种刑事犯罪，增强人民群众的安全感。加强“五边”建设，维护边境稳定和国家安全。畅通群众利益诉求渠道，认真解决信访问题。完善应急预案，有效预防、妥善处置突发事件。

　　三、加强政府自身建设

　　把对人民负责作为政府工作的第一准则、第一追求，深化效能革新、管理创新，提高行政施政能力，建设执行力较强、服务本领较高、工作作风过硬、人民群众满意的政府。

　　坚持科学理政、依法行政。发扬民主，集思广益，善于理性思考，把握矛盾变化规律，预测未来发展趋势，深入推动思想解放，努力增强决策的科学性和工作的创造性。贯彻落实市委的决策部署，自觉接受市人大的法律监督、工作监督和市政协的民主监督，认真办理人大议案、代表建议和政协委员提案。落实专家咨询、社会公示和听证制度，充分发挥民主党派、工商联、无党派人士的参政议政作用，主动听取各人民团体的意见。高度重视人民群众监督和新闻舆论监督。把依法行政贯穿于政府工作全过程，严格按照法定程序和权限行使权力、履行职责，依法管理经济社会事务。规范行政执法行为，严格执法、公正执法、文明执法。

　　加强执行力建设。树立强烈的事业心、责任感，敢于负责，敢于叫真，敢于碰硬，敢于面对矛盾和问题，着重在提高应对危机能力、改革创新能力和抓工作落实能力上下功夫。强化绩效评估，各项工作都要有目标、有标准、有责任、有监督、有检查。重点工作已经落实的看效果，尚未落实的查原因，落实不力的追责任。继续组织开展“查找改”活动，全面推行精细化管理，在城市管理、社会管理、机关管理等各个方面建立健全系统科学、具体管用的长效机制。加强市长公开电话、人民建议征集、媒体市民热线等平台建设，随时随地了解群众对政府工作的评价，及时处理群众反映的问题。提高行政效能和办事效率，确保政令畅通。

　　全面提高服务水平。以为民、利民、便民为宗旨，深化行政管理体制改革，加快形成行为规范、运转协调、公正透明、快捷高效的运行机制。深化政务公开，加强政务大厅建设，精减、规范行政审批事项和工作流程。完善领导包保责任制和重点项目、重点工作跟踪服务推进机制，落实首问负责制、服务承诺制和违诺追究制，急事急办，特事特办。加强软环境建设，深入开展民主评议软环境和政行风活动，有效解决行政不作为、乱作为、慢作为和“中梗阻”问题。加强政务诚信、商务诚信、社会诚信建设，构建诚信通化。

　　进一步改进工作作风。深入基层，深入实际，深入困难多的地方，了解百姓喜怒哀乐，体察群众安危冷暖，解难题、办实事。树立正确的政绩观，克服形式主义，不作秀、不搞“政绩工程”。问政于民、问需于民、问计于民，依靠群众把为群众办的好事办好。改进文风会风，少开会、开短会，说实话、说短话。坚决从文山会海中解脱出来，把主要精力放在研究问题、谋划发展和推动工作上。勤俭办一切事情，降低行政成本，建设节约型机关。坚持从严治政，强化行政监察和审计监督，深入落实党风廉政建设责任制，扎实推进惩治和预防腐败体系建设。加强教育、管理和监督，建设一支忠于职守、勤勉敬业、求真务实、廉洁自律的公务员队伍。

　　各位代表！通化的振兴崛起，是230万人民的期盼，是时代赋予的重任。让我们在市委的坚强领导下，在市人大、市政协的监督支持下，凝聚全市人民的智慧和力量，励精图治，勇于实践，务实创新，攻坚克难，只争朝夕，奋勇向前。
